# Supplementary material for: Remodeling of dermal adipose tissue alleviates cutaneous toxicity induced by anti-EGFR therapy
Source: eLife. 2022 Mar 24;11:e72443. doi: 10.7554/eLife.72443 (PMC8947768; doi:10.7554/eLife.72443)
Supplement: Figure 3—source data 1. [file elife-72443-fig3-data1.zip › Figure 3-source data 1.pptx]

## Slide 1
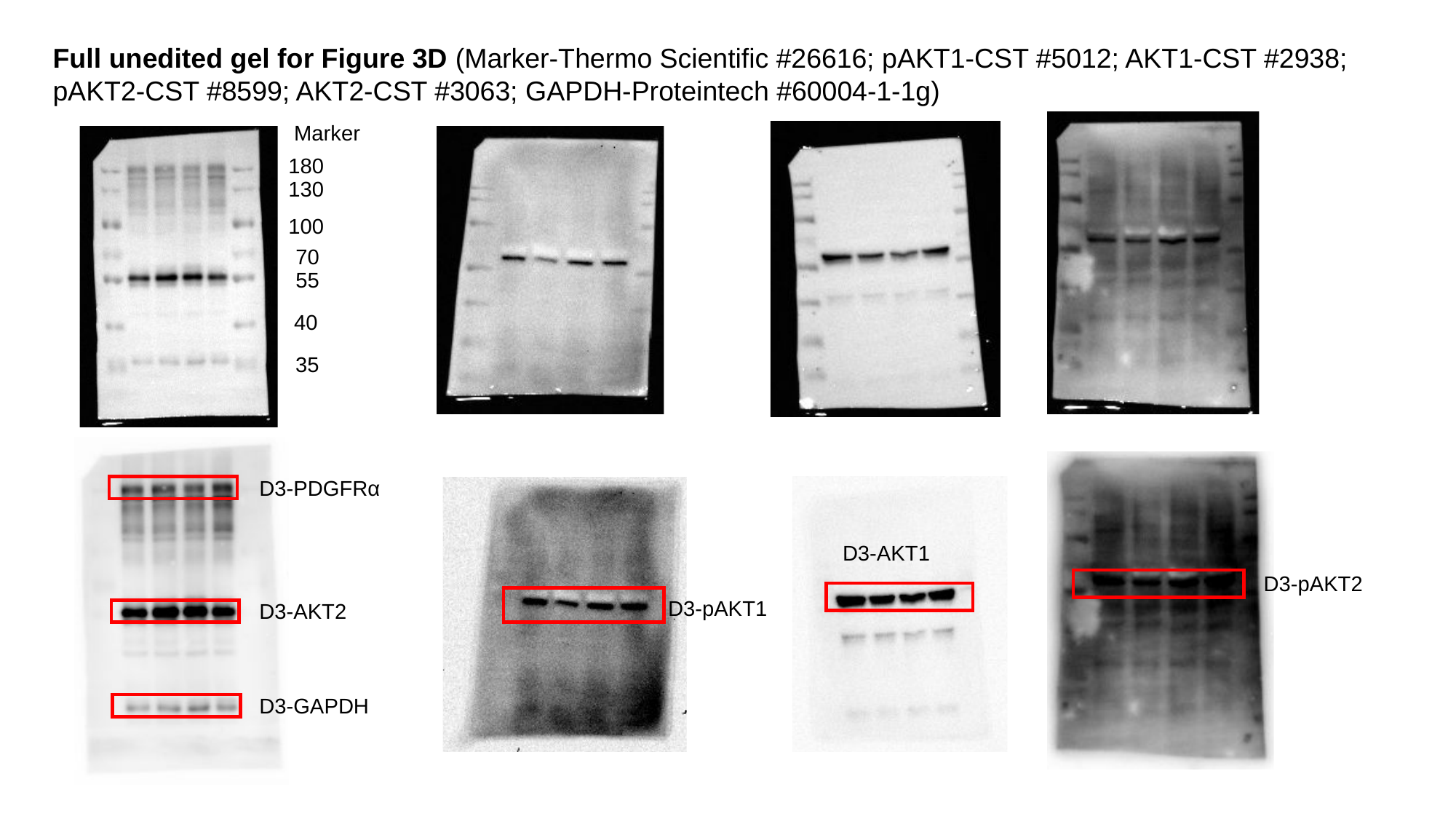

Full unedited gel for Figure 3D (Marker-Thermo Scientific #26616; pAKT1-CST #5012; AKT1-CST #2938;
pAKT2-CST #8599; AKT2-CST #3063; GAPDH-Proteintech #60004-1-1g)
Marker
180
130
100
70
55
40
35
D3-PDGFRα
D3-AKT1
D3-pAKT2
D3-pAKT1
D3-AKT2
D3-GAPDH

## Slide 2
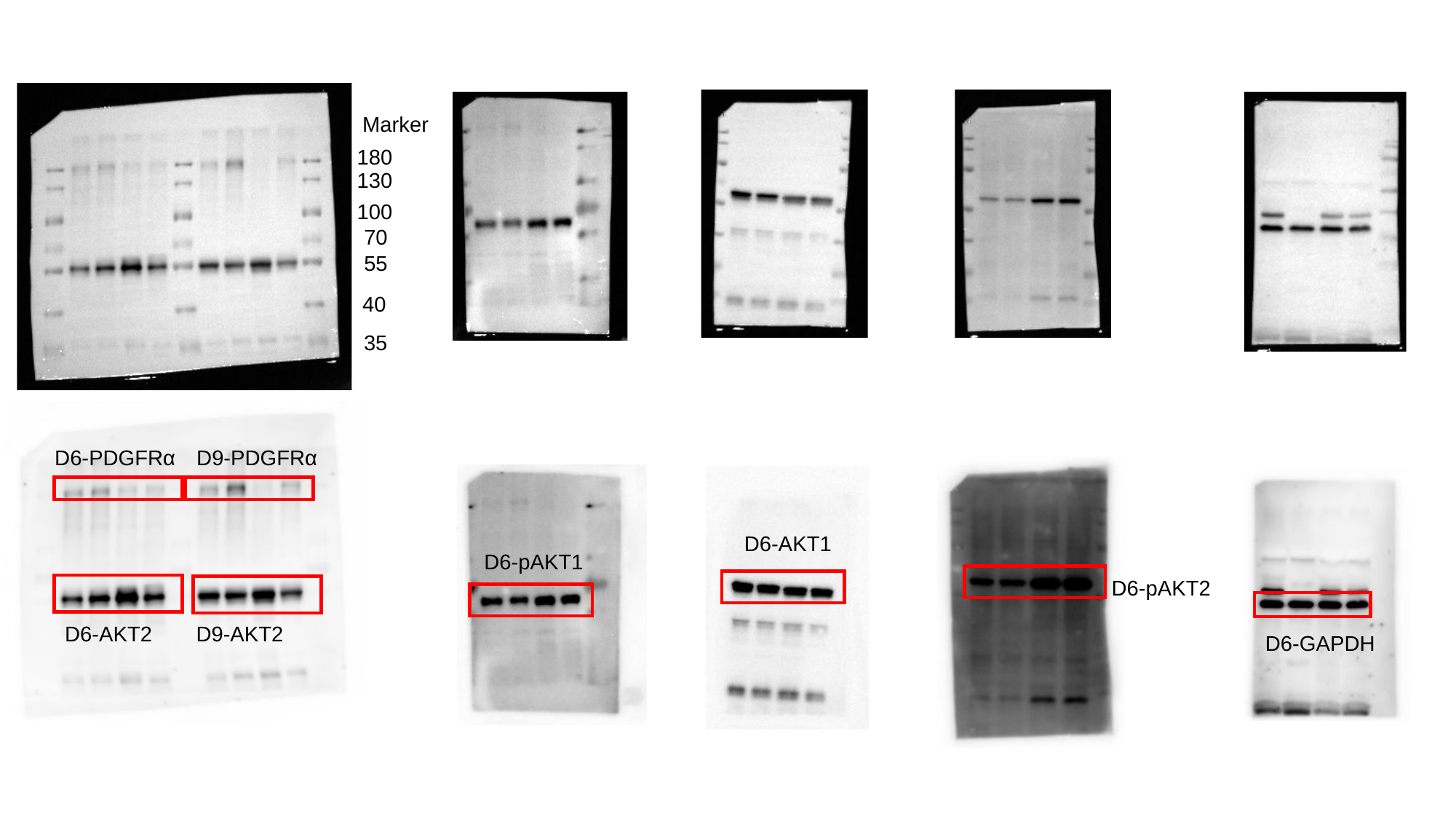

Marker
180
130
100
70
55
40
35
D9-PDGFRα
D6-PDGFRα
D6-AKT1
D6-pAKT1
D6-pAKT2
D9-AKT2
D6-AKT2
D6-GAPDH

## Slide 3
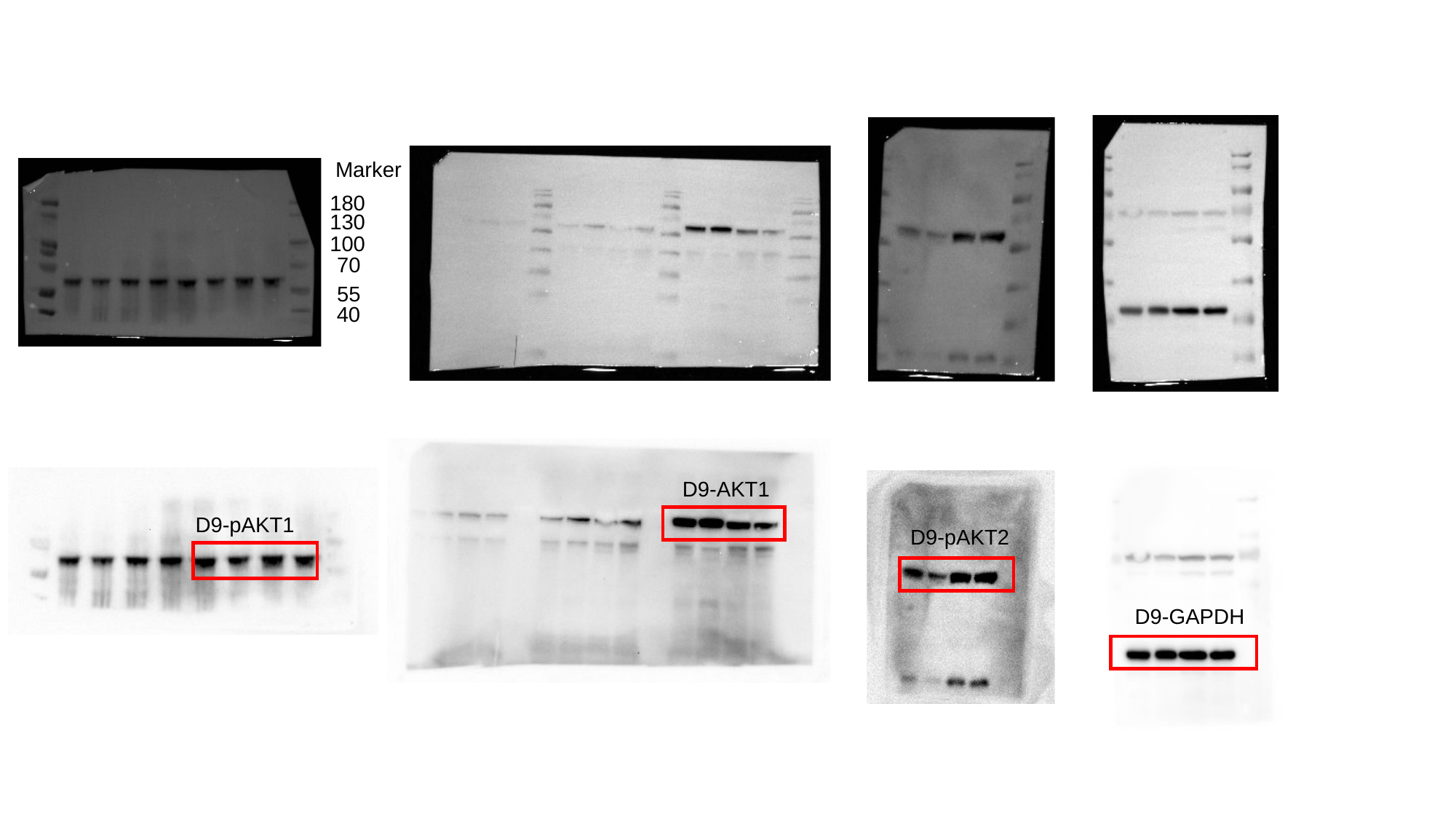

Marker
180
130
100
70
55
40
D9-AKT1
D9-pAKT1
D9-pAKT2
D9-GAPDH
