# Supplementary material for: Remodeling of dermal adipose tissue alleviates cutaneous toxicity induced by anti-EGFR therapy
Source: eLife. 2022 Mar 24;11:e72443. doi: 10.7554/eLife.72443 (PMC8947768; doi:10.7554/eLife.72443)
Supplement: Figure 4—source data 1. [file elife-72443-fig4-data1.zip › Figure 4-source data 1.pptx]

## Slide 1
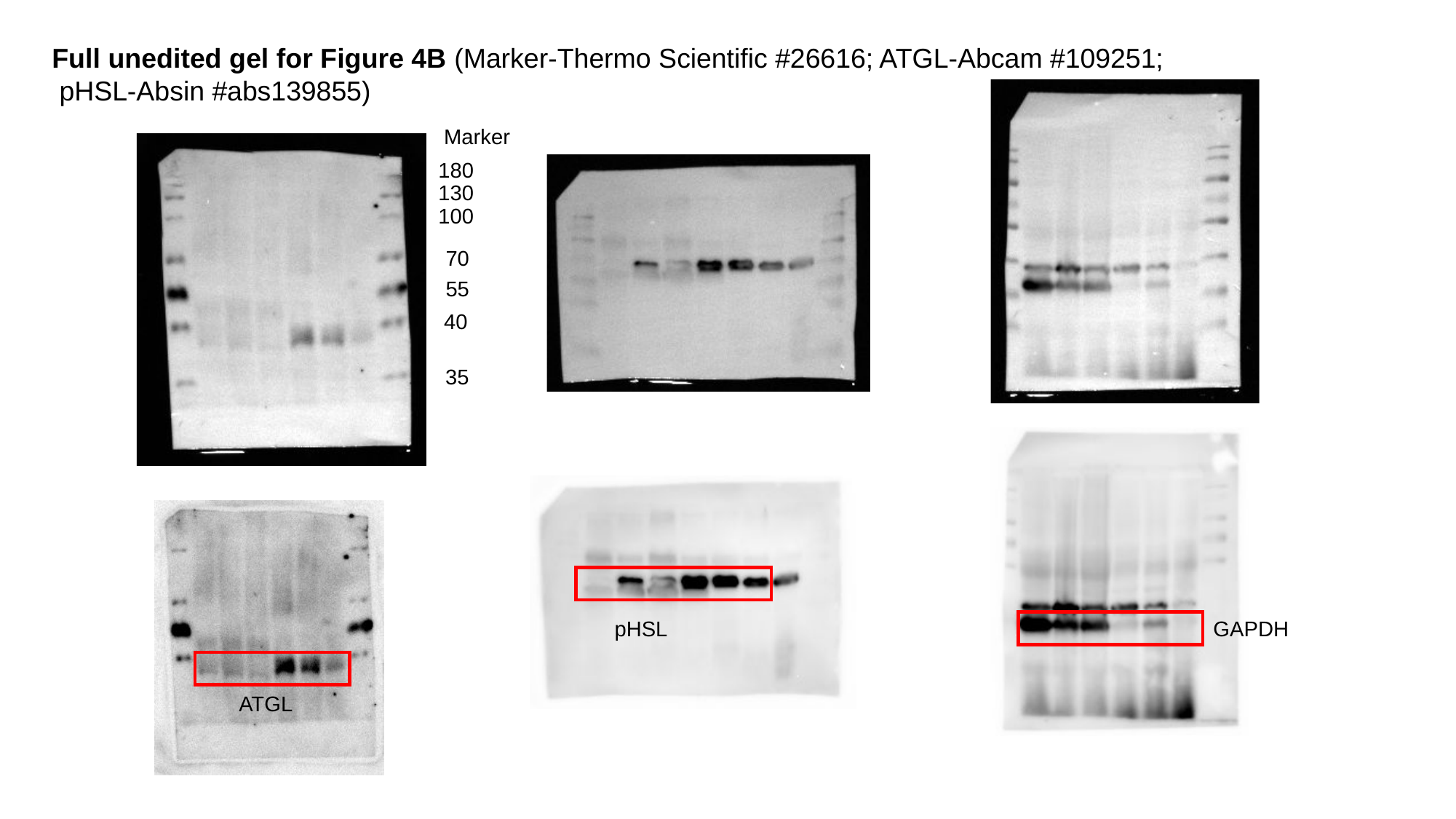

Full unedited gel for Figure 4B (Marker-Thermo Scientific #26616; ATGL-Abcam #109251;
 pHSL-Absin #abs139855)
Marker
180
130
100
70
55
40
35
pHSL
GAPDH
ATGL
