# Supplementary material for: Remodeling of dermal adipose tissue alleviates cutaneous toxicity induced by anti-EGFR therapy
Source: eLife. 2022 Mar 24;11:e72443. doi: 10.7554/eLife.72443 (PMC8947768; doi:10.7554/eLife.72443)
Supplement: Figure 4—figure supplement 1—source data 2. [file elife-72443-fig4-figsupp1-data2.zip › Figure 4-figure supplement 1-source data 2.pptx]

## Slide 1
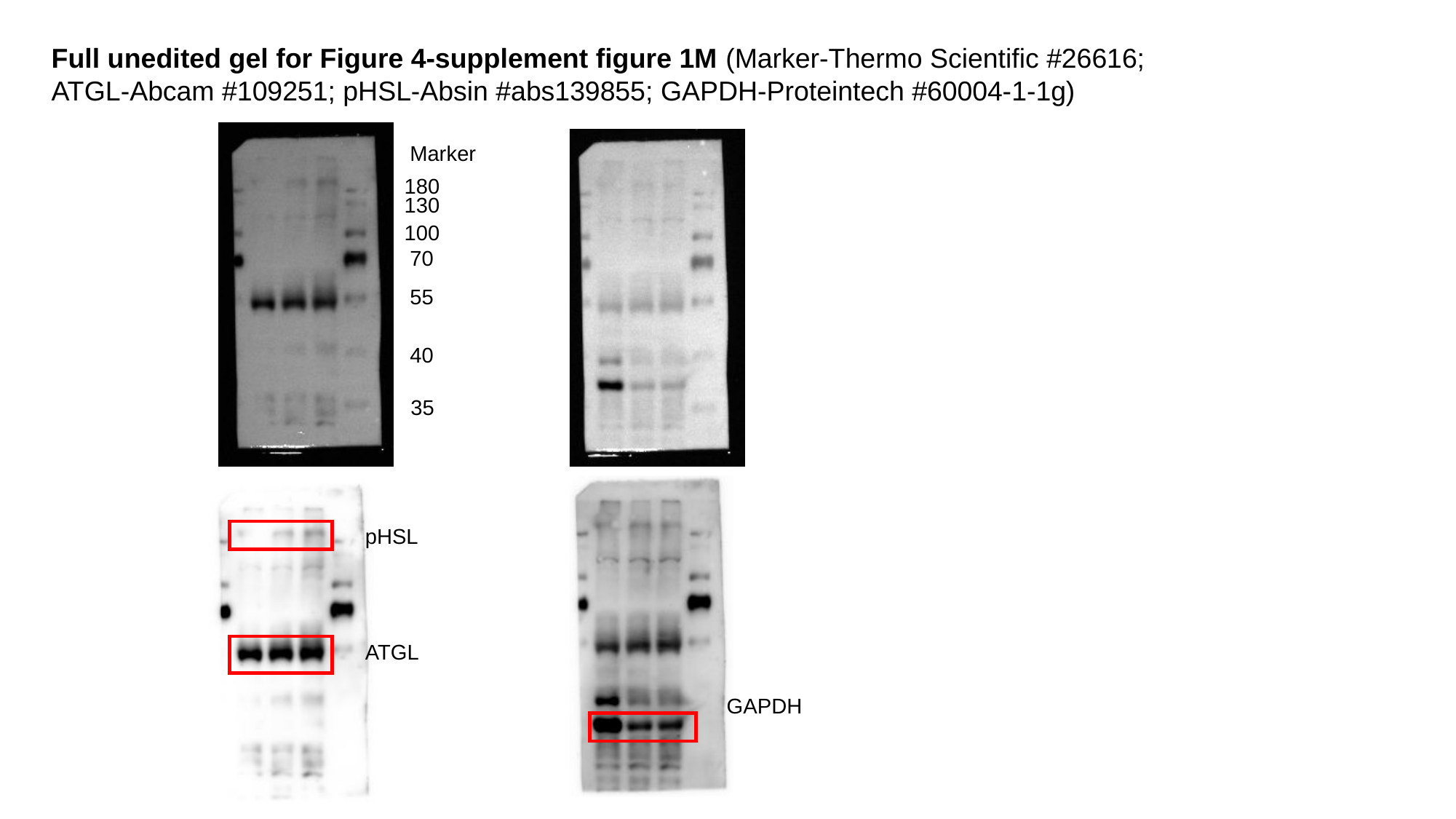

Full unedited gel for Figure 4-supplement figure 1M (Marker-Thermo Scientific #26616;
ATGL-Abcam #109251; pHSL-Absin #abs139855; GAPDH-Proteintech #60004-1-1g)
Marker
180
130
100
70
55
40
35
pHSL
ATGL
GAPDH
