# Supplementary material for: Remodeling of dermal adipose tissue alleviates cutaneous toxicity induced by anti-EGFR therapy
Source: eLife. 2022 Mar 24;11:e72443. doi: 10.7554/eLife.72443 (PMC8947768; doi:10.7554/eLife.72443)
Supplement: Figure 5—source data 1. [file elife-72443-fig5-data1.zip › Figure 5-source data 1.pptx]

## Slide 1
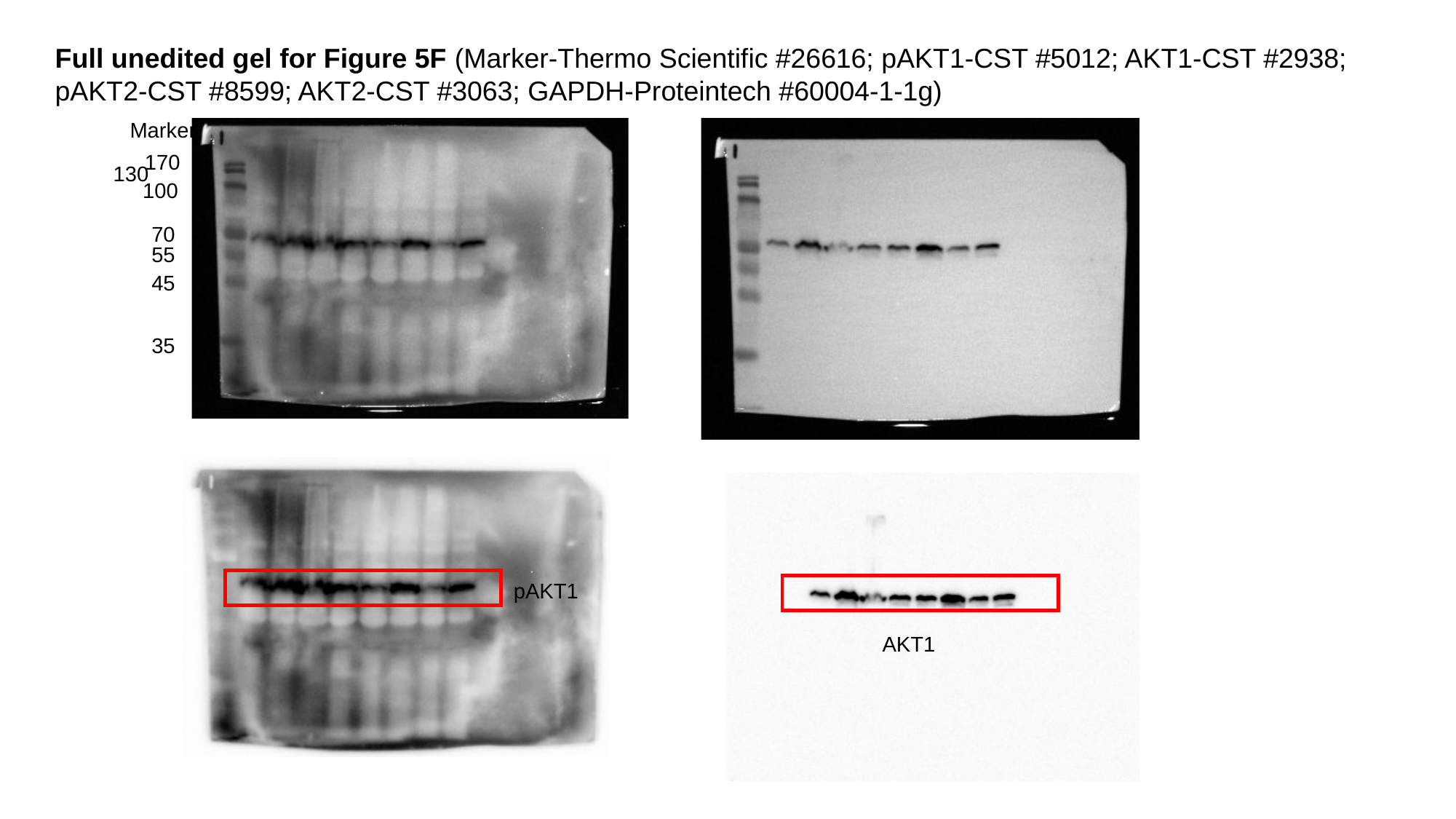

Full unedited gel for Figure 5F (Marker-Thermo Scientific #26616; pAKT1-CST #5012; AKT1-CST #2938;
pAKT2-CST #8599; AKT2-CST #3063; GAPDH-Proteintech #60004-1-1g)
Marker
170
130
100
70
55
45
35
pAKT1
AKT1

## Slide 2
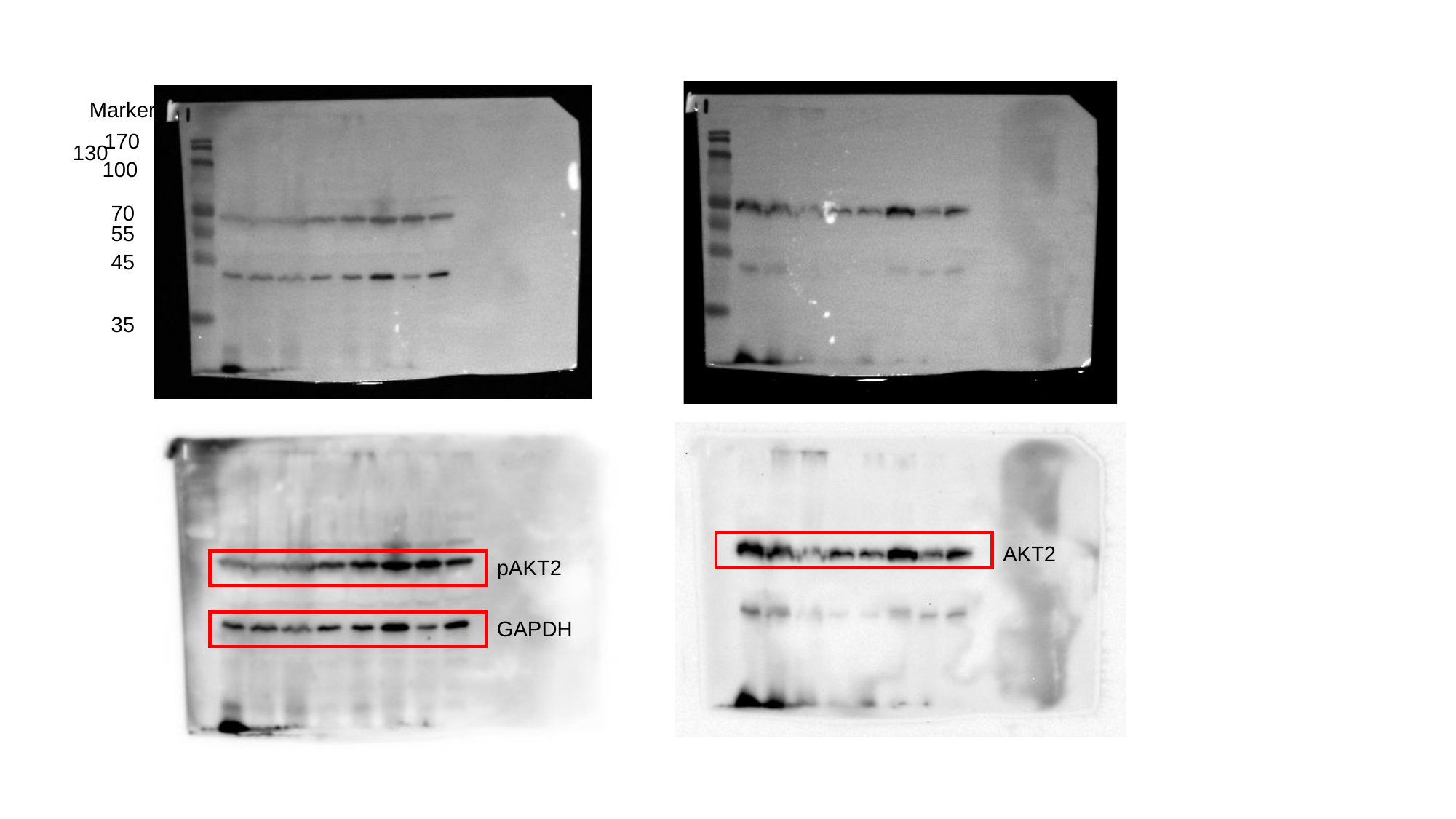

Marker
170
130
100
70
55
45
35
AKT2
pAKT2
GAPDH
