# Supplementary material for: Remodeling of dermal adipose tissue alleviates cutaneous toxicity induced by anti-EGFR therapy
Source: eLife. 2022 Mar 24;11:e72443. doi: 10.7554/eLife.72443 (PMC8947768; doi:10.7554/eLife.72443)
Supplement: Supplementary file 1. [file elife-72443-supp1.docx]

**Supplementary file 1. Weight loss in EGFRI-related clinical trials.**

| EGFR inhibitors | Trial | Weight loss (%) | |
| --- | --- | --- | --- |
|  |  | All grade | Grade≥3 |
| Afatinib | LUX-Lung 3(Sequist et al., 2013) | 17 | 1 |
| Erlotinib | SATURN(“Drug Approval Package: Tarceva (Erlotinib) NDA # 021743,” 2010) | 3.9 | ＜1 |
| Erlotinib plus gemcitabine | NCIC CTG PA.3(“Drug Approval Package: Tarceva (Erlotinib) NDA # 021743,” 2010) | 39 | 2 |
| Gefitinib | ARCHER 1050(Wu et al., 2017) | 17 | 0.4 |
| Dacomtinib | ARCHER 1050(Wu et al., 2017) | 26 | 2.2 |
| Neratinib | ExteNET(Mortimer et al., 2019) | 4.8 | 0.1 |
| Neratinib plus capecitabin | NALA(Saura et al., 2020) | 19.8 | 0.3 |
| Lapatinib plus capecitabin | NALA(Saura et al., 2020) | 13.2 | 0.6 |

**Supplemental Reference**

Drug Approval Package: Tarceva (Erlotinib) NDA # 021743. 2010. *US Food Drug Adm*.

Mortimer J, Di Palma J, Schmid K, Ye Y, Jahanzeb M. 2019. Patterns of occurrence and implications of neratinib-associated diarrhea in patients with HER2-positive breast cancer: analyses from the randomized phase III ExteNET trial. *Breast Cancer Res* **21**:32. doi:10.1186/s13058-019-1112-5

Saura C, Oliveira M, Feng Y-H, Dai M-S, Chen S-W, Hurvitz SA, Kim S-B, Moy B, Delaloge S, Gradishar W, Masuda N, Palacova M, Trudeau ME, Mattson J, Yap YS, Hou M-F, De Laurentiis M, Yeh Y-M, Chang H-T, Yau T, Wildiers H, Haley B, Fagnani D, Lu Y-S, Crown J, Lin J, Takahashi M, Takano T, Yamaguchi M, Fujii T, Yao B, Bebchuk J, Keyvanjah K, Bryce R, Brufsky A, Investigators N. 2020. Neratinib Plus Capecitabine Versus Lapatinib Plus Capecitabine in HER2-Positive Metastatic Breast Cancer Previously Treated With ≥ 2 HER2-Directed Regimens: Phase III NALA Trial. *J Clin Oncol* **38**:3138–3149. doi:10.1200/JCO.20.00147

Sequist L V., Yang JCH, Yamamoto N, O’Byrne K, Hirsh V, Mok T, Geater SL, Orlov S, Tsai CM, Boyer M, Su WC, Bennouna J, Kato T, Gorbunova V, Lee KH, Shah R, Massey D, Zazulina V, Shahidi M, Schuler M. 2013. Phase III study of afatinib or cisplatin plus pemetrexed in patients with metastatic lung adenocarcinoma with EGFR mutations. *J Clin Oncol* **31**:3327–3334. doi:10.1200/JCO.2012.44.2806

Wu Y-L, Cheng Y, Zhou X, Lee KH, Nakagawa K, Niho S, Tsuji F, Linke R, Rosell R, Corral J, Migliorino MR, Pluzanski A, Sbar EI, Wang T, White JL, Nadanaciva S, Sandin R, Mok TS. 2017. Dacomitinib versus gefitinib as first-line treatment for patients with EGFR-mutation-positive non-small-cell lung cancer (ARCHER 1050): a randomised, open-label, phase 3 trial. *Lancet Oncol* **18**:1454–1466. doi:https://doi.org/10.1016/S1470-2045(17)30608-3
