# Supplementary material for: Remodeling of dermal adipose tissue alleviates cutaneous toxicity induced by anti-EGFR therapy
Source: eLife. 2022 Mar 24;11:e72443. doi: 10.7554/eLife.72443 (PMC8947768; doi:10.7554/eLife.72443)
Supplement: Supplementary file 4. [file elife-72443-supp4.docx]

**Supplementary file 4. Primer sequence information**

| Gene | Forward primer | Reverse primer |
| --- | --- | --- |
| **Rat** |  |  |
| *Adipoq* | GTTCTCTTCACCTACGACCAGTATC | TGGTAGAGAAGGAAGCCTGTAAATG |
| *Plin2* | CTATTCTGAACCAGCCAACATCTGA | TAACTGCTCCTTTGGTCTTATCCAC |
| *Plin4* | GGATACTTCAAATTCTGCATTCCCA | GTCCTCCGTGCCTGTAAGTATATG |
| *Fabp4* | TGACAGGAAAGTGAAGAGCATC | CATGCCCTTTCGTAAACTCTTGTAG |
| *Fads2* | CATCAGCTACTATGCACGTTTCTTC | CATGACAATGTGGTTCATCTGTGTG |
| *Cd36* | AACCACTGAAGAATCTGAAGAGACC | TGAAAGCAACAAACATCACTACTCC |
| *Pparg* | CTTTATGGAGCCCAAGTTTGAGTTT | GTAGCAGGTTGTCTTGAATGTCTTC |
| *Camp* | GATGACTTCAACCAGCAGTCTTTG | CTTGCTACAGACAGTCTCCTTCAC |
| *Col1a1* | GTGGAAACCTGATGTATGCTTGAT | CTTCTGCGTCTGGTGATACATATTC |
| *Col1a2* | GCGATTACTACTGGATTGACCCTAA | CGGCTGTATGCATTCTTGGC |
| *Col5a1* | AGTGTTACCTCCAATTCCTCCAATC | GGTCAAAGTACGGGTCATAGTAGTT |
| *Col5a2* | TGACAAGATAGAGTGCCAAGAAGTG | AAGCCTTTCACCTTTCTTCCTCTAC |
| *Ctgf* | CAAGAGAATACAGGTGCCAGGAA | CAATTTGGACAAGGAGCATCAGC |
| *Atgl* | TCATCATATCGCACTTTAGCTCCAA | ACTGTGATGGTATTCTTCAGCTCAT |
| *Acta2* | CACGCTGAACTATGCTTCTGGA | CCACGCTCAGTCAGGATCTTC |
| *Pdgfra* | GATCGAAGGCAGGCACATTTATATC | TATTGTGCAAGGTTACTTCAGTGTC |
| *Lipe* | AGGGAAATAACAACTATGGAGCCAA | TCATTTGGGAGACTTTGTTTCTGTG |
| *Tgfb1* | ATAGCAACAATTCCTGGCGTTAC | CGTGGAGTACATTATCTTTGCTGTC |
| *Ccl2* | TGGAGAACTACAAGAGAATCACCAG | TCTAATGTACTTCTGGACCCATTCC |
| *Il6* | GTCATTCAGAGCAATACTGAAACCC | CAAGTGCTTTCAAGATGAGTTGGAT |
| *Tnfa*  *Il17*  *Il1b*  *Ccl2*  *Ccl5*  *Cxcl1*  *Cxcl2*  *Cxcl3*  *Ccl17*  *Ccl20* | CATGGATCTCAAAGACAACCAACTG  CCTGAAAGTCCTCAACTCCCTTAG  AATGGACAGAACATAAGCCAACAAG  TGGAGAACTACAAGAGAATCACCAG  AAGATCTCCACAGCTGCATCC  AGGGATTCACTTCAAGAACATCCAG  GGTTGACTTCAAGAACATCCAGAG  CTGAAGACCCTACCAAGGGTTG  CCACCAATGTAGGCCGAGAG  ACGTACACAAAGAACGTGTATCATC | GCTGACTTTCTCCTGGTATGAAATG  ACAGAAGGATATCTATCAGGGTCCTC  ACACAGGACAGGTATAGATTCTTCC  TCTAATGTACTTCTGGACCCATTCC  AGCACTTGCTGCTGGTGTAAA  GCTTCAGGGTCAAGGCAAGC  TTAGCCTTGCCTTTGTTCAGTATC  GGTTGAGACAAACTTCTTGACCATC  CCTTCCCTGGACAGTCTCAAAC  GGTTCTTAGGCTGAGGAGGTG |
| **Human** |  |  |
| *IL6* | AACAACCTGAACCTTCCAAAGATG | GCTTGTTCCTCACTACTCTCAAATC |
| *TNFA* | TGAGCACTGAAAGCATGATCC | ATCACTCCAAAGTGCAGCAG |
| *CCL2* | TCATAGCAGCCACCTTCATTCC | GTCTTGAAGATCACAGCTTCTTTGG |
